# *Lactiplantibacillus plantarum* BXM2 Treatment Alleviates Disorders Induced by a High-Fat Diet in Mice by Improving Intestinal Health and Modulating the Gut Microbiota

**DOI:** 10.3390/nu17030407

**Published:** 2025-01-23

**Authors:** Xiaohui Cai, Juqing Huang, Tian Yu, Xuefang Guan, Meng Sun, Dazhou Zhao, Yafeng Zheng, Qi Wang

**Affiliations:** 1 College of Food Science, Fujian Agriculture and Forestry University, Fuzhou 350002, China; cxh1231018@163.com (X.C.); yuta1010@163.com (M.S.); 2 Institute of Food Science and Technology, Fujian Academy of Agricultural Sciences, Fuzhou 350003, China; jq_huang@zju.edu.cn (J.H.); guan-619@163.com (X.G.); 3 Key Laboratory of Processing of Subtropical Characteristic Fruits, Vegetables and Edible Fungi, Ministry of Agriculture and Rural Affairs of China, Fuzhou 350002, China; 4 Bio-Fermentation Research Center, Xiamen Yuanzhidao Biotechnology Co., Ltd., Xiamen 361028, China; ytcau@163.com (T.Y.); zhao@yzdbio.com (D.Z.)

**Keywords:** obesity, probiotic, gut microbiota, cytokines, *Lactiplantibacillus plantarum* BXM2

## Abstract

**Objective:** *Lactiplantibacillus plantarum* BXM2 is a novel probiotic derived from fermented passion fruit (*Passiflora edulis*) juice that possesses promising probiotic potential. The aim of this study was to evaluate the beneficial effects of *L. plantarum* BXM2 supplementation in mice. **Methods:** *L. plantarum* BXM2 was orally administered to male SPF C57BL/6J mice fed a high-fat diet (HFD) to evaluate its anti-obesity potential, as well as the effects on intestinal health and microbiota. **Results:** Our results demonstrated that *L. plantarum* BXM2 significantly decreased the perirenal adipose index and improved intestinal health by increasing the ratio of villus height to crypt depth and the goblet cell number in the intestine. Furthermore, *L. plantarum* BXM2 treatment exhibited regulatory effects on intestinal chronic inflammation in mice by normalizing the mRNA expression of TNF-α and IL-6. Of note, *L. plantarum* BXM2 reversed HFD-induced gut dysbiosis, as evidenced by the decreased ratio of *Bacillota* (*Firmicutes*) to *Bacteroidota*, the decreased abundance of obesity-related genera *Dubosiella*, *Romboutsia*, and *Lachnospiraceae_UCG006*, and the increased abundance of beneficial genera *Akkermansia* and *Lactobacillus*. **Conclusions:** Our findings support the beneficial role of *L. plantarum* BXM2 supplementation in interventions targeting gut dysbiosis and obesity-related disorders.

## 1. Introduction

Obesity, a common metabolic disease, is becoming a major health issue worldwide. Several factors such as unhealthy eating habits, physical inactivity, and a lack of sleep are strongly associated with an increased risk of obesity [1]. According to the World Obesity Atlas 2024, the proportion of global population with overweight or obesity (BMI ≥ 25 kg/m^2^) was 38% in 2020, and it is estimated to alarmingly increase to 51% by 2034, which will cause a global economic burden amounting to USD 4 trillion [2]. In the previous decade, no country, including developing countries with a lower income, has recorded a decline in obesity prevalence across their entire population. Obesity not only negatively affects quality of life but has also been associated with chronic inflammation in metabolic tissues. Increasing evidence has indicated that obesity can induce changes in intestinal immunity and affect the gut microbiota, intestinal barrier functions, and gut-residing innate and adaptive immune cells [3,4]. Accordingly, obesity has been linked to various chronic diseases, such as hyperlipidemia, cardiovascular disease, type 2 diabetes, and certain cancers [5]. Thus, reducing the high prevalence of overweight and obesity is of great significance for public health. Among the effective therapeutic approaches for alleviating overweight and obesity, dietary interventions to improve intestinal microbiota homeostasis and intestinal health are becoming promising anti-obesity strategies [6].

Evidence has demonstrated that the onset and progression of obesity and related metabolic disorders are correlated to alterations in both intestinal microbiota function and composition, which play a crucial role in regulating host energy acquisition and appetite [7]. Compared to healthy subjects, microbial diversity and richness were significantly decreased in the obese population. The intentional manipulation of the gut microbiota by the supplementation of probiotics and prebiotics is becoming a promising strategy for anti-obesity efforts [8,9]. For example, Liu et al. demonstrated that *Lactobacillus paracasei* 24 reduced lipid accumulation in an obese mouse model by regulating the gut microbiota [10]. Notably, the stability of the gut microbiota and its metabolites is also critical for maintaining intestinal epithelial barrier integrity and shaping the intestinal immune system [11].

Probiotics are living microorganisms that have an excellent adhesion ability to colonize and proliferate in the gut, inhibiting the growth of intestinal pathogenic bacteria, thereby improving the intestinal environment and regulating the host gut homeostasis [8]. Moreover, recent evidence indicates that probiotics also have very important effects on other types of pathogens such as viruses [12], which could play a relevant role in metabolic pathologies, including obesity [13]. *Lactiplantibacillus plantarum* (formerly *Lactobacillus plantarum*), one of the most studied probiotic bacteria, has been proven to provide anti-obesity effects through balancing the gut microbiota and regulating inflammatory and immune responses. Using fluorescently labeled *L. plantarum* strains and whole-body in vivo imaging, Salomé-Desnoulez et al. discovered that orally administered *L. plantarum* mainly colonized in the intestinal lumen and, sometimes, in the crypts [14]. Ma et al. found that the gavage treatment of *L. plantarum* significantly reduced the expression of pro-inflammatory factors and increased the short-chain fatty acid (SCFA) contents in the colon, as well as reversed the intestinal flora disorder of obese mice, increasing the abundance of *Bacteroides* and *Bifidobacteriales* while reducing the abundance of *Firmicutes* and *Clostridiales* [15]. Moreover, another research indicated that *L. plantarum* HF02 ameliorated hepatic lipid accumulation by activating the adenosine monophosphate (AMP)-activated protein kinase (AMPK) pathway and ameliorated the intestinal microbiota composition by increasing the abundance of beneficial bacteria, including *Muribaculaceae*, *Akkermansia*, *Faecalibaculum*, and *Rikenellaceae_RC9_gut_group* [16].

Evidence demonstrates that probiotics counteract obesity through strain-specific mechanisms of action. Therefore, discovering more probiotics with anti-obesity effects contributes to the development of precision approaches for obesity prevention and treatment. Our previous study indicated that a novel *Lactiplantibacillus plantarum* BXM2, derived from fermented fruit juice, exhibited excellent inhibitory activity on pathogenic bacteria and survivability in gastric and intestinal conditions in vitro [17]. Hence, we speculated that *L. plantarum* BXM2 could colonize and proliferate in the colon and effectively inhibit the growth of pathogenic bacteria, promoting intestinal health and alleviating obesity-related disorders. Nevertheless, whether *L. plantarum* BXM2 exerts positive effect on obesity and intestinal health in an animal model is still unclear, as its mechanism remains to be validated.

To bridge this knowledge gap, this study evaluated the protective effects of *L. plantarum* BXM2 in mice fed a high-fat diet (HFD). Initially, the effects of *L. plantarum* BXM2 on body weight, adiposity index, organ index, and intestinal morphology were evaluated. Then, we analyzed the effects of *L. plantarum* BXM2 on the expression of immune cytokines and barrier function-associated genes. Finally, the regulatory effects of *L. plantarum* BXM2 on the intestinal microbiota were verified.

## 2. Materials and Methods

### 2.1. Materials and Reagents

A normal chow diet (4.3% fat by wt., 10% kcal, no. H10010) and a high-fat diet (35% fat by wt., 60% kcal, no. H10060) for the experimental mice were customized by Beijing Huafukang Bioscience Co., Inc. (Beijing, China). The MRS (de Man, Rogosa and Sharpe) medium was purchased from Beijing Land Bridge Technology (Beijing, China). SweScript RT I First Strand cDNA Synthesis Kit was purchased from Servicebio (Wuhan, China). The TruSeqTM DNA Sample Prep Kit and AxyPrep DNA Gel Recovery Kit were obtained from Axygen Biosciences (Hangzhou, China). Other reagents were analytically pure.

### 2.2. Preparation of the Lactiplantibacillus plantarum BXM2 Strain

*Lactiplantibacillus plantarum* BXM2 (patent number: ZL201910682492.X) was isolated from a naturally fermented passion fruit (*Passiflora edulis*) honey beverage in the Fujian province, China, and stored in the Chinese General Microorganism Collection Center (CGMCC NO.16436). The strain, obtained from the Fujian Academy of Agricultural Sciences (Fuzhou, China), was grown in MRS medium at 37 °C for 24 h, centrifuged at 4 °C, 5000× *g* for 10 min, and washed three times with 0.9% sterile saline buffer. The cells were resuspended in saline buffer at a concentration of 1 × 10^10^ CFU/mL. Fresh bacterial suspensions were prepared daily for gavage feeding.

### 2.3. Animals and Experimental Design

Animal experiments were conducted in accordance with the guidelines and approved by the Institutional Animal Care and Use Committee of the Fujian Agriculture and Forestry University (approval code: PZCASFAFU24106). All efforts were made to minimize animal suffering. A total of 21 male SPF C57BL/6J mice (4–5 weeks old) were purchased from Wu’s Laboratory Animal Co. (Fuzhou, China) and housed at the Experimental Animal Center under the following controlled conditions: a 12 h light/dark cycle, 25 ± 1 °C room temperature, and 55 ± 5% relative humidity. The mice had unlimited access to water and food.

After a week of adaptation, the mice were randomly divided into three groups (n = 8), as follows: the control group (CON), fed with chow diet and 0.2 mL normal saline by gavage; the model group (HFD), fed with HFD and 0.2 mL normal saline by gavage; and the BXM2-treated group (BXM2), fed with HFD and 0.2 mL *L. plantarum* BXM2 suspension by gavage. During a six-week period of intragastric administration, the food intake and body weight of the mice were measured and recorded weekly. After the feeding period, all mice were fasted for 16 h and sacrificed with diethyl ether. An autopsy was performed to collect the samples, including perirenal and epididymal adipose tissues and organs (liver, spleen, thymus, and intestine), for further study. The cecal contents were aseptically collected, placed in sterile cryotubes, immediately frozen with liquid nitrogen, and stored at −80 °C for intestinal microbiota analysis.

### 2.4. Evaluation of Adiposity Index and Organ Index

The total weight of the perirenal and epididymal adipose tissues was measured to evaluate the visceral fat mass. For calculating the adiposity index (mg/g), the adipose tissue weight (mg) was divided by the body weight (g).

Liver, spleen, and thymus were stripped and weighed to determine the liver index and the immune organ (spleen and thymus) index. For calculating the organ index (mg/g), the organ weight (mg) was divided by the body weight (g).

### 2.5. Histological Analysis

Intestinal segments corresponding to the jejunum (the middle part of the small intestine) and the ileum (the distal part of the small intestine) were prepared for histological analysis as described previously [18]. The samples were cleaned with PBS buffer, and soaked in 4% (*w*/*v*) paraformaldehyde (pH 7.0) for fixation. After dehydration, paraffin wax was soaked and embedded to make 5 μm slices, which were stained with hematoxylin–eosin (H&E) or periodic acid–Schiff (PAS). After staining, the slices were sealed and observed by a biological microscope (Olympus BX53, Olympus Corporation, Tokyo, Japan) equipped with a digital camera (Eclipse Ci-L; Nikon, Tokyo, Japan). The crypt depth and villus height of the jejunum and ileum were measured using the Image-Pro Plus 6.0 software (Media Cybernetics, Rockville, MD, USA). Additionally, PAS staining was used to detect the number of intestinal goblet cells in the jejunum.

### 2.6. Quantitative Real-Time Reverse Transcription–Polymerase Chain Reaction (qRT-PCR) Analysis

Total RNA of the jejunum samples was extracted using the NucleoZol reagent (Gene Company, Shanghai, China), according to the manufacturer’s protocol. After the determination of RNA purity and concentration, the total RNA was reversely transcribed into cDNA. The expression levels of IL-6, TNF-α, *E*-cadherin, and occludin were determined with Ultra SYBR Mixture (Cowin Biotech, Beijing, China) on the qPCR Detection System (HealForce CG-05, Shanghai, China), with β-actin as the internal reference gene. The primer sequences used are listed in Table 1. The expression of mRNA was calculated according to the 2^−ΔΔCt^ method, and the relative expression levels were presented as fold changes compared to the CON group.

### 2.7. Gut Microbiota Genomic DNA Extraction and 16S rRNA Sequencing

Genomic DNA extraction from cecal samples was performed using the Fast DNA SPIN Extraction Kit (MP Biomedicals, Irvine, CA, USA), and the total DNA concentration and purity were characterized by 1% agarose gel electrophoresis. Following DNA extraction, 16S rRNA sequencing was carried out to assess the microbial diversity and composition. The V3-V4 variable region of the bacterial 16S rRNA gene was amplified by an ABI GeneAmp 9700 PCR thermocycler (ABI, Los Angeles, CA, USA) with the universal primer sets 338F (5′-ACTCCTACGGGAGGCAGAG-3′) and 806R (5′-GGACTA CHVGGGTWTCTAAT-3′). Subsequently, the amplicons were purified and quantified to ensure accurate library preparation. Purified amplicons were pooled in equimolar concentrations and sequenced on an Illumina MiSeq platform (Illumina, San Diego, CA, USA) according to the standard protocols provided by Majorbio Bio-Pharm Technology Co., Ltd. (Shanghai, China).

Quantitative Insights into Microbial Ecology (QIIME, version 1.9.0) was used to analyze the amplicon data. Amplicon sequence variant (ASV) identification was performed to classify the sequences into taxonomic units. The assignment of taxonomic identities to the generated ASVs was accomplished through comparison with the reference database (SILVA, version 138). Subsequently, based on ASV representative sequences and abundance information, a bioinformatic analysis was performed on the Majorbio cloud platform (https://cloud.majorbio.com, accessed on 10 May 2024).

### 2.8. Statistical Analysis

All data were measured independently and in parallel at least 3 times, and the data results were expressed as the mean ± standard deviation. Origin 9.0 software was used for plotting. SPSS 24.0 software was used for statistical analysis of the experimental data, and the significant difference (*p* < 0.05) analysis was conducted by a one-way variance analysis (ANOVA) followed by Turkey’s test.

## 3. Results and Discussion

### 3.1. Effect of L. plantarum BXM2 on Body Weight

**Figure 1:**
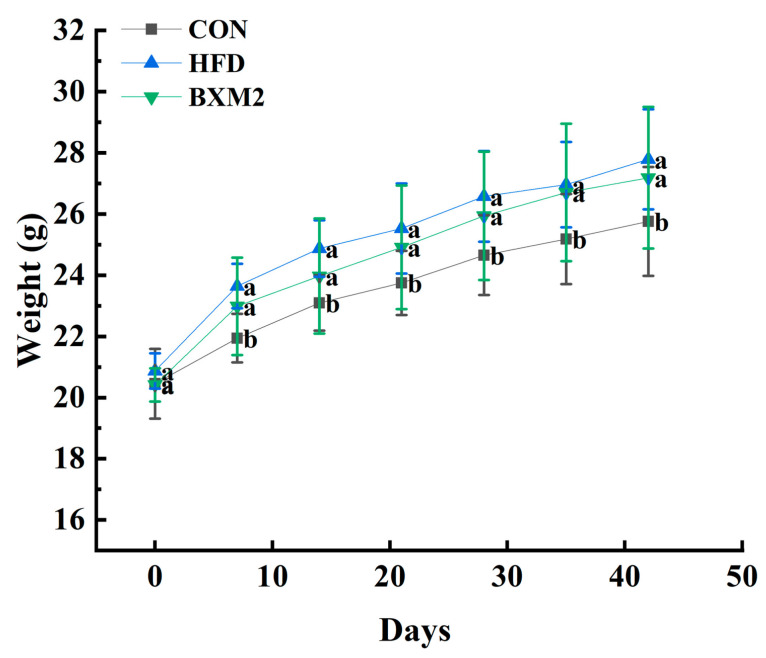
Effect of *Lactiplantibacillus plantarum* BXM2 on body weight in mice fed a high-fat diet. CON: chow diet control group; HFD: high-fat diet group; and BXM2: HFD + *Lactiplantibacillus plantarum* BXM2 treatment group. Different superscript letters indicate statistical differences among groups (*p* < 0.05).

During the animal experimental period, no mortality was observed, and no obvious abnormalities in animal appearance, behavior, and food intake were noted. As shown in Figure 1, compared to the CON group, the remaining two groups fed a high-fat diet exhibited a significant increase in body weight (*p* < 0.05), indicating that obesity was successfully induced in mice. On the sixth week, the group treated with *L. plantarum* BXM2 showed a slightly lower body weight than the HFD group, but the difference was not statistically significant. This may have been due to the continuous feeding of mice with an HFD during the intervention period, which inevitably induced the eventual occurrence of obesity. Moreover, it has been reported that not all *Lactobacillus* strains are able to directly reduce body weight in obese mice [19]. For example, supplementation with *Lactobacillus acidophilus* NCDC 13 [20] or *Lactobacillus casei* Shirota [21] did not affect body weight in obese mice during the experimental period but was still able to alleviate disorders induced by a high-fat diet, delaying the progression of obesity.

### 3.2. Effect of L. plantarum BXM2 on Adiposity Index and Organ Index

**Figure 2:**
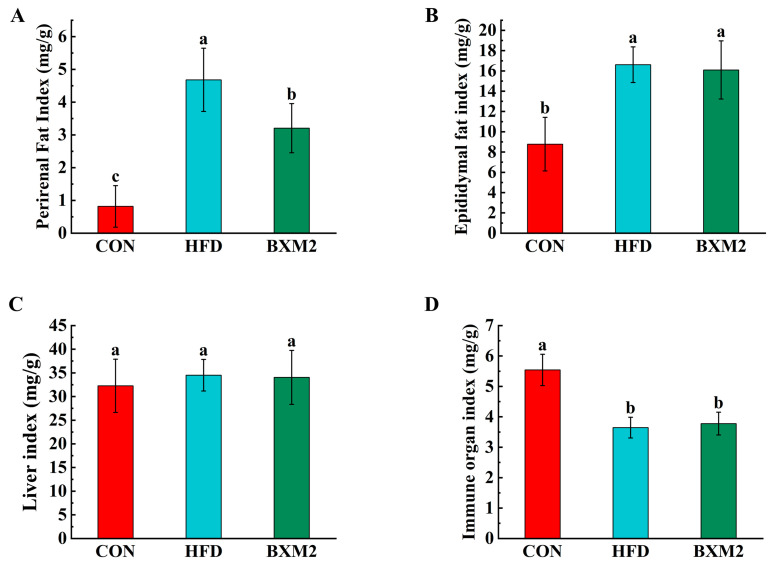
Effect of *Lactiplantibacillus plantarum* BXM2 on adiposity index (**A**,**B**) and organ index (**C**,**D**) in mice fed a high-fat diet. CON: chow diet control group; HFD: high-fat diet group; and BXM2: HFD + *Lactiplantibacillus plantarum* BXM2 treatment group. Different superscript letters indicate statistical differences among groups (*p* < 0.05).

Compared to the CON group, there was a significant increase in the perirenal fat and epididymal fat indexes in the HFD group (Figure 2A,B). It was notable that the intervention employing *L. plantarum* BXM2 significantly decreased the perirenal fat index compared to the HFD group (*p* < 0.05). However, *L. plantarum* BXM2 supplementation did not significantly affect the epididymal fat index in obese mice (*p* > 0.05). These results suggest that *L. plantarum* BXM2 has the potential to alleviate perirenal fat deposition in obese mice.

The organ index is one of the indicators used to measure the health of mice. As shown in Figure 2C, both high-fat diet induction and *L. plantarum* BXM2 intervention did not cause any notable difference in the liver index of mice (*p* > 0.05). Compared to CON group, the immune organ (spleen and thymus) index of obese mice was significantly decreased (*p* < 0.05), which indicated the immunosuppression resulting from the high-fat diet (Figure 2D). After the intervention employing *L. plantarum* BXM2, the immune organ index of high-fat diet mice was not significantly changed (*p* > 0.05), which might have been due to the short intervention time.

### 3.3. Effect of L. plantarum BXM2 on Intestinal Morphology

**Figure 3:**
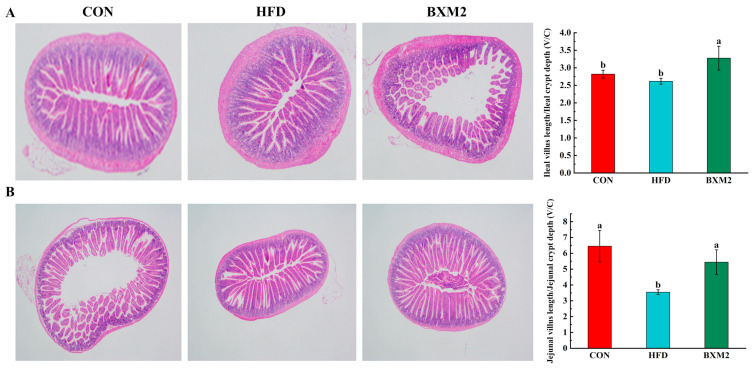
Effect of *Lactiplantibacillus plantarum* BXM2 on the ratio of villus height to crypt depth in the ileum (**A**) and jejunum (**B**) of mice fed a high-fat diet. CON: chow diet control group; HFD: high-fat diet group; and BXM2: HFD + *Lactiplantibacillus plantarum* BXM2 treatment group. Different superscript letters indicate statistical differences among groups (*p* < 0.05).

Hematoxylin–eosin (H&E) staining was performed on the ileum and jejunum tissues of mice in each group. According to the images of the target area, five intact intestinal villus heights (from the top to the base of the villus) and their associated crypt depths (from the base of the villus to the bottom of the crypt) in each section were measured with millimeters as the standard unit (Figure 3). The ratio of villus height to crypt depth (V/C) was calculated to reflect intestinal health and functional status [22]. The mice in the HFD group exhibited a slight decrease in the V/C ratio in the ileum (*p* > 0.05) and a significant decline in the V/C ratio in the jejunum (*p* < 0.05), compared to those in the CON group. Conversely, the intervention employing *L. plantarum* BXM2 notably increased the V/C ratios in both the ileum and jejunum tissues of obese mice (*p* < 0.05).

**Figure 4:**
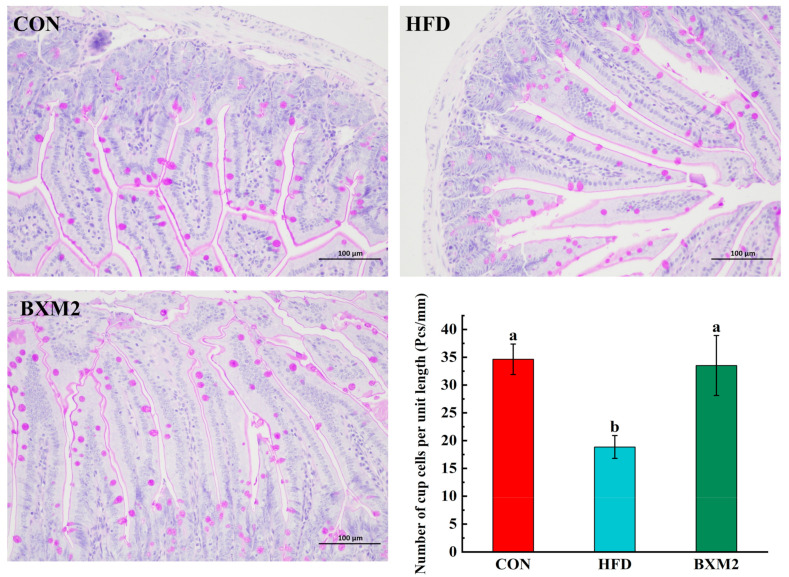
Effect of *Lactiplantibacillus plantarum* BXM2 on jejunum goblet cells in mice fed a high-fat diet. CON: chow diet control group; HFD: high-fat diet group; and BXM2: HFD + *Lactiplantibacillus plantarum* BXM2 treatment group. Different superscript letters indicate statistical differences among groups (*p* < 0.05).

The PAS-stained sections of the jejunum were observed under a microscope, and the number of goblet cells per unit length was calculated. As shown in Figure 4, the number of goblet cells per unit length in the jejunum of mice in the HFD group was significantly lower than in the CON group (*p* < 0.05), indicating a reduction in jejunum goblet cells in mice during high-fat diet feeding. However, interventions employing *L. plantarum* BXM2 could significantly increase the number of goblet cells in the jejunum of mice fed a high-fat diet (*p* < 0.05). Intestinal goblet cells have an essential role in maintaining intestinal health through providing a protective mucus barrier and regulating the gut’s immune responses [23]. A prior study reported a similar result that the administration of *Lactobacillus plantarum* increased the number of goblet cells in the intestine [24]. The findings from the intestinal morphology analysis demonstrate that supplementation of *L. plantarum* BXM2 can increase the V/C ratio and the goblet cell number in obese mice, which contribute to intestinal integrity, promote a healthier intestinal mucosal layer, and are ideal for maintaining gut immune homeostasis.

### 3.4. Effect of L. plantarum BXM2 on the Expression of Immune Cytokines and Barrier Function-Associated Genes

**Figure 5:**
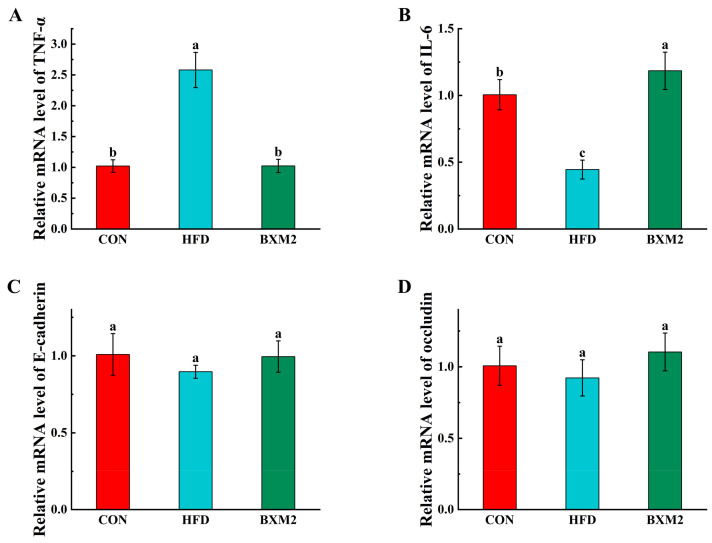
Effect of *Lactiplantibacillus plantarum* BXM2 on the mRNA expression of TNF-α (**A**), IL-6 (**B**), *E*-cadherin (**C**), and occludin (**D**) in mice fed a high-fat diet. CON: chow diet control group; HFD: high-fat diet group; and BXM2: HFD + *Lactiplantibacillus plantarum* BXM2 treatment group. Different superscript letters indicate statistical differences among groups (*p* < 0.05).

High-fat diet consumption has been noted to alter intestinal barrier function and intestinal chronic inflammation [3]. To evaluate whether treatment with *L. plantarum* BXM2 could modulate the levels of inflammatory cytokines, the expression levels of IL-6 and TNF-α in the jejunum tissue were measured (Figure 5A,B). It is commonly known that obesity is a state of chronic low-grade inflammation, associated with increased levels of TNF-α and IL-6 in the plasma and adipose tissues [25,26]. However, in response to HFD feeding, we found that the TNF-α mRNA level was clearly upregulated, while the expression of IL-6 was downregulated in the HFD group compared to the control animals (*p* < 0.05). IL-6 is an interleukin that acts as a pro-inflammatory cytokine but can also have anti-inflammatory activities as a myokine. Therefore, HFD feeding may impair innate immunity due to the damage of intestinal immune cells, leading to a reduction in IL-6 expression. It has been reported in a previous study that *Limosilactobacillus fermentum* may enhance innate immunity by upregulating IL-6 and IL-10 at the RNA level in HT-29 cells [27]. Significantly, treatment with *L. plantarum* BXM2 reversed the changes induced by the HFD on the expression of inflammatory cytokines (*p* < 0.05). The expression of inflammatory genes in mice fed an HFD could be time- and location-specific [28]. To further understand the complex mechanism of the immunomodulatory effects, it would be important to repeat such analyses from other segments of the bowel at different time points in future animal experiments.

Occludin and *E*-cadherin play an important role in the formation and maintenance of the intestinal epithelial barrier. Diet-induced obesity is associated with increased intestinal permeability and reduced expression of intercellular junction proteins [3]. In this study, the mice fed an HFD also exhibited moderately lower mRNA expression of occludin and *E*-cadherin in jejunum tissues, although no significant differences were reached when compared with the CON group (Figure 5C,D). Increasing evidence suggests that probiotic administration may contribute to maintaining the integrity of the epithelial barrier, which is essential for establishing a healthy intestinal environment. For example, *Lactobacillus rhamnosus* GG can exhibit protective effects on the intestinal mucosa from pepsin–trypsin–digested gliadin (PTG)-induced damage by preventing the reduction in the expression of intercellular junction proteins [29]. The downregulation of the expression of occludin and *E*-cadherin due to HFD feeding might be reversed by *L. plantarum* BXM2 administration, but no significant differences were achieved in our study between the HFD and BXM2 groups after the six-week experiment.

### 3.5. Effect of L. plantarum BXM2 on the Gut Microbiota

#### 3.5.1. Changes in Gut Microbial Richness and Diversity

**Figure 6:**
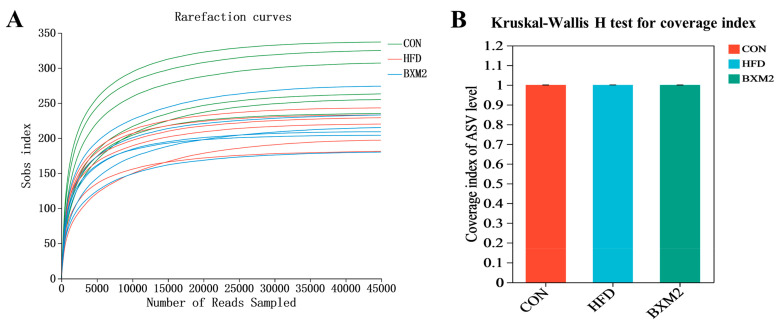
Sequencing data quality assessment: (**A**) colonic microbial dilution curve for mouse intestinal contents; and (**B**) coverage index. CON: chow diet control group; HFD: high-fat diet group; and BXM2: HFD + *Lactiplantibacillus plantarum* BXM2 treatment group.

The gut microbiota is closely associated with obesity, and dysregulation of the gut microbial community affects many metabolic functions. To investigate the influence of *L. plantarum* BXM2 treatment on gut microbial composition, the cecal contents from different groups were collected and sent for 16S rRNA sequencing. The sequencing data quality was assessed using the dilution curve and the coverage index. As shown in Figure 6A, the dilution curve of each sample eventually tends to be flat, indicating reasonable and representative sequencing data. Meanwhile, the coverage index is very close to 1, suggesting that the sequencing depth essentially covered all bacterial communities in the samples (Figure 6B).

**Figure 7:**
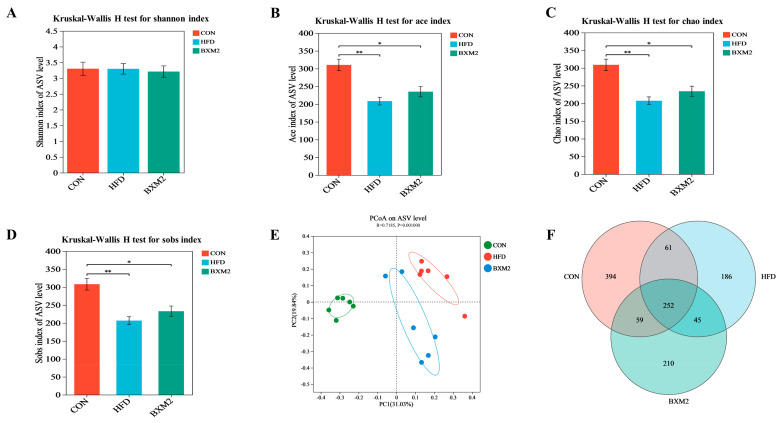
Effect of *Lactiplantibacillus plantarum* BXM2 on gut microbial α diversity: (**A**) Shannon index; (**B**) Ace index; (**C**) Chao index; (**D**) Sobs index; (**E**) principal coordinate (PCoA) analysis; and (**F**) Venn diagram analysis. CON: chow diet control group; HFD: high-fat diet group; and BXM2: HFD + *Lactiplantibacillus plantarum* BXM2 treatment group. Significant differences are indicated as * for *p* < 0.05 and ** for *p* < 0.01.

Alpha diversity is mainly used to evaluate community diversity in the samples, and different indexes can be analyzed to obtain indicators of species diversity and richness in communities. Figure 7A–D display the α diversity indexes of the intestinal microbial community in three groups, in which the Chao index, the Sobs index, and the Ace index represent species richness, while the Shannon index represents species diversity. Compared to the CON group, the Chao index, the Sobs index, and the Ace index of the intestinal microbial community in the HFD group significantly decreased (*p* < 0.01), while the Shannon index showed no significant changes (*p* > 0.05). These results indicate that a high-fat diet could significantly reduce the species richness of the intestinal microbial community in mice but has no significant effect on the species coverage and diversity. As shown by the Chao, Sobs, and Ace indexes, the intervention employing *L. plantarum* BXM2 significantly increased the gut microbial community richness in the mice fed an HFD. Our results are consistent with those reported in a recent research that investigated the beneficial effects of *L. plantarum* HF02 in obese mice [16].

A Beta diversity analysis reflects the inter-group differences among different communities and was analyzed using principal coordinate (PCoA) analysis in this study. The PCoA result showed an obvious separation of the intestinal flora structure among the CON, HFD, and BXM2 groups (Figure 7E): the closer the distance reflected in the PCoA diagram, the greater the similarity of the flora structure between the samples. It is clear that the *L. plantarum* BXM2 treatment improved the intestinal flora structure in the mice fed an HFD.

A Venn diagram was used to analyze the similarity and specificity of species distribution among samples in each group (Figure 7E). The shared ASVs between the three groups were 252. Meanwhile, 394 and 186 unique ASVs were identified in the CON group and the HFD group, suggesting that HFD feeding reduced both species diversity and species specificity in the mouse intestine. With the *L. plantarum* BXM2 intervention, the unique ASVs increased to 210, revealing that probiotic intervention restored the species richness of the intestine in the mice fed an HFD.

#### 3.5.2. Changes in the Composition of Gut Microbiota

**Figure 8:**
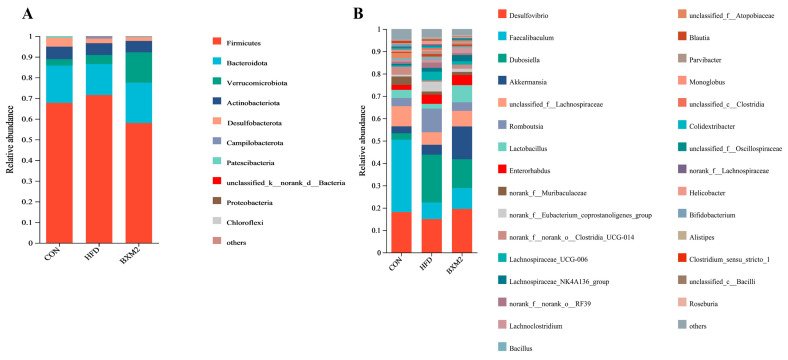
Effect of *Lactiplantibacillus plantarum* BXM2 on the relative abundance of microbial at the phylum (**A**) and genus (**B**) level in mice. CON: chow diet control group; HFD: high-fat diet group; and BXM2: HFD + *Lactiplantibacillus plantarum* BXM2 treatment group.

The relative abundance of intestinal microorganisms in the mice of each group at the phylum level is presented as a stacking histogram in Figure 8A. The intestinal microbes of the mice in each group were mainly composed of *Bacillota* (*Firmicutes*), *Bacteroidota*, *Verrucomicrobiota*, *Actinomycetota* (*Actinobacteriota*), and *Thermodesulfobacteriota* (*Desulfobacterota*) at the phylum level. *Firmicutes* and *Bacteroidota* were the two main phyla of bacteria, accounting for more than 80% of the microbiota of the mice in each group. Existing studies have shown that an increase in *Firmicutes* and a decrease in *Bacteroidota* are strongly associated with the development of obesity [9,30].

**Figure 9:**
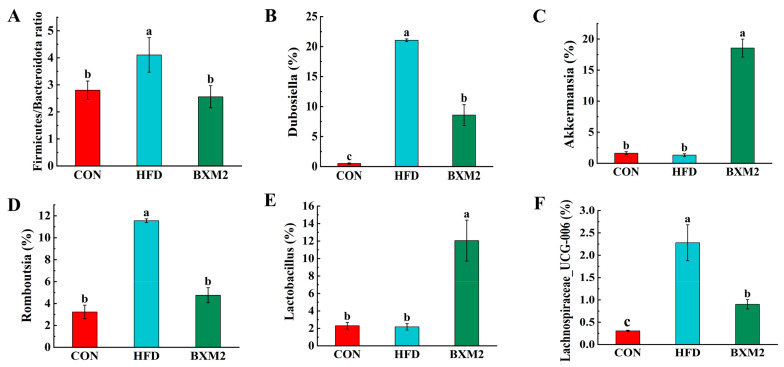
Effect of *Lactiplantibacillus plantarum* BXM2 on gut microbial composition in mice: *Firmicutes*/*Bacteroidota* ratio (**A**) and relative abundance of five significantly altered bacterial genera, including *Dubosiella* (**B**), *Akkermansia* (**C**), *Romboutsia* (**D**), *Lactobacillus* (**E**), and *Lachnospiraceae_UCG-006* (**F**). CON: chow diet control group; HFD: high-fat diet group; and BXM2: HFD + *Lactiplantibacillus plantarum* BXM2 treatment group. Different superscript letters indicate statistical differences among groups (*p* < 0.05).

Compared to the CON group, the relative abundance of *Firmicutes* increased from 67.72% to 71.51% in the HFD group, while the relative abundance of *Bacteroidota* decreased from 18.07% to 14.96% (Figure 8A), resulting in an increase in the *Firmicutes*/*Bacteroidota* ratio (Figure 9A, *p* < 0.05). *L. plantarum* BXM2 treatment reversed these changes in the composition of the gut microbiota in mice fed an HFD and significantly decreased the *Firmicutes*/*Bacteroidota* ratio (Figure 9A, *p* < 0.05), indicating that *L. plantarum* BXM2 may play a crucial role in alleviating the gut dysbiosis caused by an HFD. It is notable that the relative abundance of *Verrucomicrobiota* in the BXM2 group increased to 3.33-fold compared to the HFD group (Figure 8A). The phylum *Verrucomicrobia* is known for its high abundance of short-chain fatty acid (SCFA)-producing and mucin-degrading potential probiotics, including, especially, *Akkermansia muciniphila* [31].

Previous studies have indicated that probiotics positively affect the structure of the gut microbiota through improving the relative abundance of beneficial bacteria and reducing the proportion of harmful bacteria, playing a positive role in alleviating obesity. Liu et al. found that *Lactobacillus paracasei* 24 treatment regulated the abundance and diversity of the gut microbiota in HFD-induced obese mice, reduced the abundance of *Firmicutes* and the ratio of *Firmicutes*/*Bacteroidota*, and increased the abundance of *Akkermansia* [10]. Cai et al. investigated the gut microbiota of obese mice treated with *Lactobacillus plantarum* FRT4, isolated from a local yogurt. The results confirmed that probiotic administration can reshape the gut microbiota by increasing the relative abundance of *Bacteroidota* and decreasing the number of *Firmicutes* [1].

The relative abundance of intestinal microorganisms in the mice from each group was analyzed at the genus level, and the distribution of the top 30 bacterial genera with the highest abundance was summarized, as shown in Figure 8B. *Faecalibaculum*, *Desulfovibrio*, *unclassified_f__Lachnospiraceae*, *norank_f__Muribaculaceae*, and *Lactobacillus* were the most abundant bacterial genera in the CON group. HFD feeding induced changes in the microbiota, and *Dubosiella*, *Desulfovibrio*, *Romboutsia*, *Faecalibaculum*, and *unclassified_f__Lachnospiraceae* became the most abundant bacterial genera in the HFD group. In the BXM2 group, the genera with higher relative abundance were *Akkermansia*, *Dubosiella*, *Desulfovibrio*, *Faecalibaculum*, and *Lactobacillus*. Compared to the CON group, the relative abundance of *Faecalibaculum* in the HFD group was notably decreased, indicating that a high-fat diet exhibited a great impact on the growth of beneficial bacteria of the *Faecalibaculum* genus [32]. Compared to the HFD group, the administration of *L. plantarum* BXM2 increased the relative abundance of *Faecalibaculum*, but the difference was not statistically significant. Meanwhile, a high-fat diet resulted in the significant increases in the relative abundance of *Dubosiella* (Figure 9B), *Romboutsia* (Figure 9D), and *Lachnospiraceae_UCG-006* (Figure 9F), respectively (*p* < 0.05). In this study, *L. plantarum* BXM2 treatment dramatically reversed the increases in these bacterial genera (*p* < 0.05). Of note, compared to both the CON and HFD groups, the relative abundance of *Akkermansia* (Figure 9C) and *Lactobacillus* (Figure 9E) in the BXM2 group significantly increased (*p* < 0.05).

Previous studies have shown that several bacterial genera are strongly correlated with obesity development. For example, Bai et al. indicated that *Dubosiella* was significantly increased in mice fed an HFD [33]. Qiu et al. identified that *Dubosiella* was associated with HFD feeding-induced gut dysbiosis and negatively associated with SCFA production [34]. According to a pilot-scale cross-sectional study in Indonesia, *Romboutsia* was found to be abnormally increased in an obese group, with a dysbiosis-like microbiota community, and it was identified as an obesity-related genus correlated to lipid profiles and lipogenesis in the liver [35]. Forte et al. discovered that *Lachnospiraceae_UCG006*, a genus positively associated with obesity and food addiction, was found to be significantly increased in the large intestine of mice fed a high-fat diet [36]. Therefore, these genera, including *Dubosiella*, *Romboutsia*, and *Lachnospiraceae_UCG006*, might be harmful gut microorganisms associated with the development of obesity and other diseases. However, more in-depth studies still need to be conducted to prove the strong correlation between these gut microbes and obesity. In our study, *L. plantarum* BXM2 intervention reversed the significant increase in the relative abundance of *Dubosiella*, *Romboutsia,* and *Lachnospiraceae_UCG006* caused by a high-fat diet, which is consistent with the conclusions from the above studies.

Of note, *L. plantarum* BXM2 intervention dramatically boosted the relative abundance of bacterial genera such as *Akkermansia* and *Lactobacillus*. *Akkermansia* is a typical genus of beneficial bacteria, and its representative strain is *Akkermansia muciniphila*, which has gradually been considered a promising candidate for next-generation probiotics. In various animal and human studies, physiological benefits of *Akkermansia* have been found, including the ability to reduce inflammation and oxidative damage, lower the serum cholesterol levels [37], and restore intestinal barrier function [10,38]. Combined with our results from intestinal morphology and the expression of barrier function-associated genes, it is suggested that an HFD might destroy intestinal barrier integrity in mice, while an increased abundance of *Akkremansia* might play an important role in restoring intestinal barrier function. In a recent study carried out in Sweden, significantly lower levels of *Akkermansia* and *Desulfovibrio* were confirmed in preschool children with excessive body weight [39]. Supplementation with probiotics or prebiotics has been proven to exhibit beneficial effects by promoting the growth of *Akkermansia* in the gut [38,40]. The *Lactobacillus* genus is another well-known beneficial gut bacterium, which can improve gut health and exhibit anti-inflammatory, anti-diabetic, and anti-obesity effects [41]. Khan et al. investigated the probiotic effects of *L. plantarum* strains in the mouse colitis model and discovered that *L. plantarum* strains improved dysbiosis and significantly enhanced the growth of bacterial genera *Akkermansia* and *Lactobacillus* [42]. In summary, *L. plantarum* BXM2 treatment does not significantly reduce the bodyweight of mice fed an HFD, but it can regulate the composition and diversity of the gut microbiota in these mice and thus alleviate disorders related to obesity.

## 4. Conclusions

Our results suggest that *L. plantarum* BXM2 supplementation has positive regulatory effects on HFD-induced disorders in mice. *L. plantarum* BXM2 significantly reduced the perirenal adipose index in mice and increased the ratio of villus height to crypt depth and the goblet cell number in the intestine, which are ideal for maintaining gut immune homeostasis. Furthermore, *L. plantarum* BXM2 treatment counteracted HFD-induced intestinal chronic inflammation in obese mice by normalizing the mRNA expression of TNF-α and IL-6. In addition, *L. plantarum* BXM2 treatment reversed HFD-induced gut dysbiosis, as indicated by the reduction in the *Firmicutes*-to-*Bacteroidetes* ratio, the decrease in obesity-related genera *Dubosiella*, *Romboutsia*, and *Lachnospiraceae_UCG006*, and the increase in beneficial genera *Akkermansia* and *Lactobacillus*. Our findings support the beneficial role of *L. plantarum* BXM2 supplementation for interventions targeting gut dysbiosis and obesity-related disorders. Further investigation is needed to understand the probiotic mechanism of *L. plantarum* BXM2, promoting its potential as a novel probiotic with anti-obesity functions.

## Figures and Tables

**Table 1:** Primer sequences used in real-time PCR.

Target Genes	Forward Primer	Reverse Primer
IL-6	CTGCAAGAGACTTCCATCCAG	AGTGGTATAGACAGGTCTGTTGG
TNF-α	GCCGATGGGTTGTACCTTGT	TCTTGACGGCAGAGAGGAGG
*E*-cadherin	CAGGTCTCCTCATGGCTTTGC	CTTCCGAAAAGAAGGCTGTC
Occludin	ATGGCTGCTGCTGATGAATA	CTTGATGTGCGATAATTTGCTCTT
β-actin	ACTGCCGCATCCTCTTCCTC	AAAGAGCCTCAGGGCATCGG

## Data Availability

The original contributions presented in the study are included in the article, further inquiries can be directed to the corresponding author.

## References

[1] Cai H., Wen Z., Zhao L., Yu D., Meng K., Yang P. (2022). Lactobacillus plantarum FRT4 alleviated obesity by modulating gut microbiota and liver metabolome in high-fat diet-induced obese mice. Food Nutr. Res..

[2] World Obesity Federation World Obesity Atlas 2024. https://data.worldobesity.org/publications/WOF-Obesity-Atlas-v7.pdf.

[3] Winer D.A., Luck H., Tsai S., Winer S. (2016). The intestinal immune system in obesity and insulin resistance. Cell Metab..

[4] Cani P.D., Jordan B.F. (2018). Gut microbiota-mediated inflammation in obesity: A link with gastrointestinal cancer. Nat. Rev. Gastroenterol. Hepatol..

[5] Karlsson E.A., Sheridan P.A., Beck M.A. (2010). Diet-induced obesity impairs the t cell memory response to influenza virus infection. J. Immunol..

[6] Mio K., Otake N., Nakashima S., Matsuoka T., Aoe S. (2021). Ingestion of high beta-glucan barley flour enhances the intestinal immune system of diet-induced obese mice by prebiotic effects. Nutrients.

[7] Schütz F., Figueiredo-Braga M., Barata P., Cruz-Martins N. (2021). Obesity and gut microbiome: Review of potential role of probiotics. Porto Biomed. J..

[8] Cheng Z., Zhang L., Yang L., Chu H. (2022). The critical role of gut microbiota in obesity. Front. Endocrinol..

[9] Gangzheng W., Xianglian C., Chengyuan S., Qiuju H., Chenghua Z., Min L., Jianping X., Xueshuang H., Wangqiu D. (2023). Gut microbiota and metabolite insights into anti-obesity effect of carboxymethyl pachymaran in high-fat diet mice. J. Funct. Foods.

[10] Liu Z., Zhou X., Wang W., Gu L., Hu C., Sun H., Xu C., Hou J., Jiang Z. (2022). *Lactobacillus paracasei* 24 attenuates lipid accumulation in high-fat diet-induced obese mice by regulating the gut microbiota. J. Agric. Food Chem..

[11] Wei S., Wang L., Chen X., Wang Y., Tong L., Han Q., Ren B., Guo D. (2024). Anti-inflammatory activity of boletus aereus polysaccharides: Involvement of digestion and gut microbiota fermentation. Food Chem. X.

[12] Lopez-Santamarina A., Lamas A., Del Carmen Mondragón A., Cardelle-Cobas A., Regal P., Rodriguez-Avila J.A., Miranda J.M., Franco C.M., Cepeda A. (2021). Probiotic effects against virus infections: New weapons for an old war. Foods.

[13] Ezzatpour S., Mondragon Portocarrero A.D.C., Cardelle-Cobas A., Lamas A., López-Santamarina A., Miranda J.M., Aguilar H.C. (2023). The human gut virome and its relationship with nontransmissible chronic diseases. Nutrients.

[14] Salome-Desnoulez S., Poiret S., Foligne B., Muharram G., Peucelle V., Lafont F., Daniel C. (2021). Persistence and dynamics of fluorescent *Lactobacillus plantarum* in the healthy versus inflamed gut. Gut Microbes.

[15] Ma Y., Fei Y., Han X., Liu G., Fang J. (2022). *Lactobacillus plantarum* alleviates obesity by altering the composition of the gut microbiota in high-fat diet-fed mice. Front. Nutr..

[16] Chen H., Zhao H., Qi X., Sun Y., Ma Y., Li Q. (2023). *Lactobacillus plantarum* hf02 alleviates lipid accumulation and intestinal microbiota dysbiosis in high-fat diet-induced obese mice. J. Sci. Food Agr..

[17] Guan X., Zhao D., Yang Y., Huang J., Lin B., Zheng Y., Wang Q. (2023). Characterization and in vitro assessment of probiotic potential of *Lactiplantibacillus plantarum* bxm2 from fermented honey passion fruit beverage. Food Front..

[18] Tao W., Wang G., Pei X., Sun W., Wang M. (2022). Chitosan oligosaccharide attenuates lipopolysaccharide-induced intestinal barrier dysfunction through suppressing the inflammatory response and oxidative stress in mice. Antioxidants.

[19] Dao M.C., Everard A., Clément K., Cani P.D. (2016). Losing weight for a better health: Role for the gut microbiota. Clin. Nutr. Exp..

[20] Arora T., Anastasovska J., Gibson G., Tuohy K., Sharma R.K., Bell J., Frost G. (2012). Effect of *Lactobacillus acidophilusncdc* 13 supplementation on the progression of obesity in diet-induced obese mice. Brit. J. Nutr..

[21] Naito E., Yoshida Y., Makino K., Kounoshi Y., Kunihiro S., Takahashi R., Matsuzaki T., Miyazaki K., Ishikawa F. (2011). Beneficial effect of oral administration of *Lactobacillus casei* strain shirota on insulin resistance in diet-induced obesity mice. J. Appl. Microbiol..

[22] Liu Y., He Y., Fan S., Gong X., Zhou Y., Jian Y., Ouyang J., Jiang Q., Zhang P. (2023). Effects of led light colors on the growth performance, intestinal morphology, cecal short-chain fatty acid concentrations and microbiota in broilers. Animals.

[23] Gustafsson J.K., Johansson M.E.V. (2022). The role of goblet cells and mucus in intestinal homeostasis. Nat. Rev. Gastroenterol. Hepatol..

[24] Zeng Z., Huang Z., Yue W., Nawaz S., Chen X., Liu J. (2023). *Lactobacillus plantarum* modulate gut microbiota and intestinal immunity in cyclophosphamide-treated mice model. Biomed. Pharmacother..

[25] Schneider-Matyka D., Cybulska A.M., Szkup M., Pilarczyk B., Panczyk M., Lubkowska A., Sadowska N., Grochans E. (2023). Selenium as a factor moderating depression and obesity in middle-aged women. Nutrients.

[26] Lee A.Y., Christensen S.M., Duong N., Tran Q.A., Xiong H.M., Huang J., James S., Vallabh D., Talbott G., Rose M. (2022). Sirt3 pharmacologically promotes insulin sensitivity through pi3/akt/mtor and their downstream pathway in adipocytes. Int. J. Mol. Sci..

[27] Prakash V., Madhavan A., Veedu A.P., Babu P., Jothish A., Nair S.S., Suhail A., Prabhakar M., Sain T., Rajan R. (2023). Harnessing the probiotic properties and immunomodulatory effects of fermented food-derived *Limosilactobacillus fermentum* strains: Implications for environmental enteropathy. Front. Nutr..

[28] Leon I.C., Quesada-Vazquez S., Sainz N., Guruceaga E., Escote X., Moreno-Aliaga M.J. (2020). Effects of maresin 1 (mar1) on colonic inflammation and gut dysbiosis in diet-induced obese mice. Microorganisms.

[29] Orlando A., Linsalata M., Bianco G., Notarnicola M., D'Attoma B., Scavo M.P., Tafaro A., Russo F. (2018). *Lactobacillus rhamnosus* gg protects the epithelial barrier of wistar rats from the pepsin-trypsin-digested gliadin (ptg)-induced enteropathy. Nutrients.

[30] Yanez C.M., Hernandez A.M., Sandoval A.M., Dominguez M.A.M., Muniz S.A.Z., Gomez J.O.G. (2021). Prevalence of blastocystis and its association with firmicutes/bacteroidetes ratio in clinically healthy and metabolically ill subjects. BMC Microbiol..

[31] Pinitchun C., Panpetch W., Bhunyakarnjanarat T., Udompornpitak K., Do H.T., Visitchanakun P., Wannigama D.L., Udomkarnjananun S., Sukprasansap M., Tencomnao T. (2024). Aging-induced dysbiosis worsens sepsis severity but is attenuated by probiotics in d-galactose-administered mice with cecal ligation and puncture model. PLoS ONE.

[32] Liu Y., Huang W., Ji S., Wang J., Luo J., Lu B. (2022). *Sophora japonica* flowers and their main phytochemical, rutin, regulate chemically induced murine colitis in association with targeting the nf-kappab signaling pathway and gut microbiota. Food Chem..

[33] Bai Y.F., Wang S.W., Wang X.X., Weng Y.Y., Fan X.Y., Sheng H., Zhu X.T., Lou L.J., Zhang F. (2019). The flavonoid-rich quzhou fructus aurantii extract modulates gut microbiota and prevents obesity in high-fat diet-fed mice. Nutr. Diabetes.

[34] Qiu X., Macchietto M.G., Liu X., Lu Y., Ma Y., Guo H., Saqui-Salces M., Bernlohr D.A., Chen C., Shen S. (2021). Identification of gut microbiota and microbial metabolites regulated by an antimicrobial peptide lipocalin 2 in high fat diet-induced obesity. Int. J. Obesity.

[35] Therdtatha P., Song Y., Tanaka M., Mariyatun M., Almunifah M., Manurung N.E.P., Indriarsih S., Lu Y., Nagata K., Fukami K. (2021). Gut microbiome of indonesian adults associated with obesity and type 2 diabetes: A cross-sectional study in an asian city, yogyakarta. Microorganisms.

[36] Forte N., Roussel C., Marfella B., Lauritano A., Villano R., De Leonibus E., Salviati E., Khalilzadehsabet T., Giorgini G., Silvestri C. (2023). Olive oil-derived endocannabinoid-like mediators inhibit palatable food-induced reward and obesity. Commun. Biol..

[37] Higarza S.G., Arboleya S., Arias J.L., Gueimonde M., Arias N. (2021). *Akkermansia muciniphila* and environmental enrichment reverse cognitive impairment associated with high-fat high-cholesterol consumption in rats. Gut Microbes.

[38] Cheng D., Xie M.Z. (2021). A review of a potential and promising probiotic candidate-*Akkermansia muciniphila*. J. Appl. Microbiol..

[39] Karlsson C.L.J., Önnerfält J., Xu J., Molin G., Ahrné S., Thorngren-Jerneck K. (2012). The microbiota of the gut in preschool children with normal and excessive body weight. Obesity.

[40] Dong C., Yu J., Yang Y., Zhang F., Su W., Fan Q., Wu C., Wu S. (2021). Berberine, a potential prebiotic to indirectly promote akkermansia growth through stimulating gut mucin secretion. Biomed. Pharmacother..

[41] Li C., Shi S. (2023). Gut microbiota and metabolic profiles in chronic intermittent hypoxia-induced rats: Disease-associated dysbiosis and metabolic disturbances. Front. Endocrinol..

[42] Khan I., Wei J., Li A., Liu Z., Yang P., Jing Y., Chen X., Zhao T., Bai Y., Zha L. (2022). *Lactobacillus plantarum* strains attenuated dss-induced colitis in mice by modulating the gut microbiota and immune response. Int. Microbiol..

